# A Narrative Review of the Velocity and Acceleration Profile in Football: The Influence of Playing Position

**DOI:** 10.3390/sports13010018

**Published:** 2025-01-10

**Authors:** Charles Cotteret, Ángel González-de-la-Flor, Jaime Prieto Bermejo, Jaime Almazán Polo, Sergio L. Jiménez Saiz

**Affiliations:** 1 Department of Physiotherapy, Faculty of Medicine, Health and Sports, European University of Madrid, 28670 Villaviciosa de Odón, Spain; charles.cotteret@universidadeuropea.es (C.C.); angel.gonzalez@universidadeuropea.es (Á.G.-d.-l.-F.); jaime.almazan@universidadeuropea.es (J.A.P.); 2 Faculty of Economic and Business Sciences, Universidad Rey Juan Carlos, 28032 Madrid, Spain; jaime.prieto@urjc.es; 3 Sport Sciences Research Centre, Universidad Rey Juan Carlos, 28943 Fuenlabrada, Spain

**Keywords:** GPS technology, initial speed, playing positions, acceleration profile, speed running

## Abstract

To enhance athletic performance and reduce the risk of injury, load quantification has allowed for a better understanding of the individual characteristics of the physical demands on soccer players during training or competition. In this regard, it appears crucial to summarize scientific evidence to provide useful information and future directions related to the speed and acceleration profiles of male soccer players. This review aims to evaluate the findings reflected in the available literature on both profiles in football, synthesizing and discussing data from scientific articles, while providing insights into quantification methods, employed thresholds, tracking systems, terminology, playing position, and microcycle day. Therefore, it is hoped that this narrative review can support objective decision-making in practice for coaches, sports scientists, and medical teams regarding individualized load management and the appropriate selection of metrics, to explore current trends in soccer player profiles.

## 1. Introduction

### 1.1. Characteristics and Evolution of Physical Demands in Soccer, Technology

Soccer is a complex team sport influenced by a multitude of contextual variables that interact at both the player and team levels [1]. Due to this complexity, the assessment of players’ and teams’ physical, technical, and tactical abilities cannot be understood in isolation [2,3]. From a physical demand’s perspective, soccer is primarily an intermittent aerobic sport, where players alternate between high-intensity, multidirectional efforts and numerous low-intensity rest periods [4,5]. During competition, while most efforts occur at low speeds, several critical aspects of successful performance outcomes require high-speed actions [5].

The literature over the last decade provides consensus on the significant increase in high-intensity actions in soccer without an accompanying rise in the total distance covered [6,7,8,9]. Longitudinal studies over several seasons in the English and Spanish leagues show notable increases in high-intensity and sprint distances. This change in the game’s intensity is partly attributed to evolving tactical strategies that prioritize quick transitions, high pressing, and rapid counterattacks. These strategies demand more frequent high-intensity accelerations, which allow players to cover shorter distances at higher speeds. Alongside this, there are longer rest periods between actions, enabling greater recovery and sustaining a more intense style of play. This shift highlights the importance of players’ ability to perform high-intensity actions intermittently and repeatedly, a critical factor for physical performance in soccer [10,11,12,13].

### 1.2. Load Monitoring and Technology

**Figure 1:**
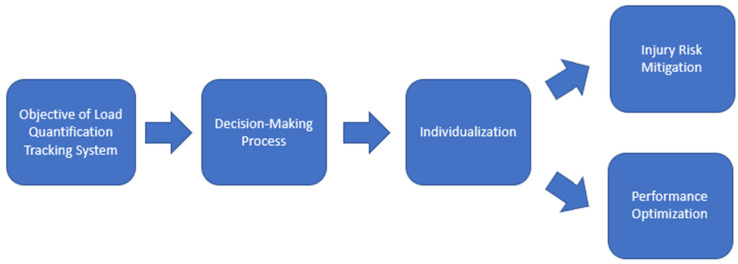
Objective of Load Quantification in Football.

Monitoring athletes in team sports is increasingly important to understand individual responses to load, with the goal of optimizing physical performance and minimizing injury risk (Figure 1) [14,15]. Individual adaptations to physical exercise can vary significantly among players and are related to individual fitness levels, as well as the intensity and duration of training loads. Therefore, it is essential to tailor training programs in an individualized manner [14,16,17,18,19]. There are various methods for load individualization, and studies show a strong interest in investigating these methods [14,20,21].

In contemporary soccer, load indicators need to be interpretable in real time so that coaches can make decisions without sacrificing training time for assessments or load monitoring [22,23].

The three most commonly used analysis devices in soccer are multi-camera optical sensor video systems, local positioning systems (LPS) based on radar, and global positioning systems (GPS) (Table 1) [18,24,25,26,27]. One of the major drawbacks, apart from the fixed and costly installation like LPS, is the inability to use these systems for real-time training monitoring. This limitation arises because LPS typically require a controlled environment with a fixed setup, making them impractical for dynamic training conditions where mobility is essential [28,29]. In the past decade, there has been an increase in the use of GPS technology, making it the most widely used system in current soccer (Table 1) [12,30,31,32]. GPS offers the possibility of objectively measuring a wide range of variables, allowing coaches to understand players’ conditioning needs according to playing position during training sessions or competitions [4,10,20,33].

### 1.3. External Load Monitoring

**Figure 2:**
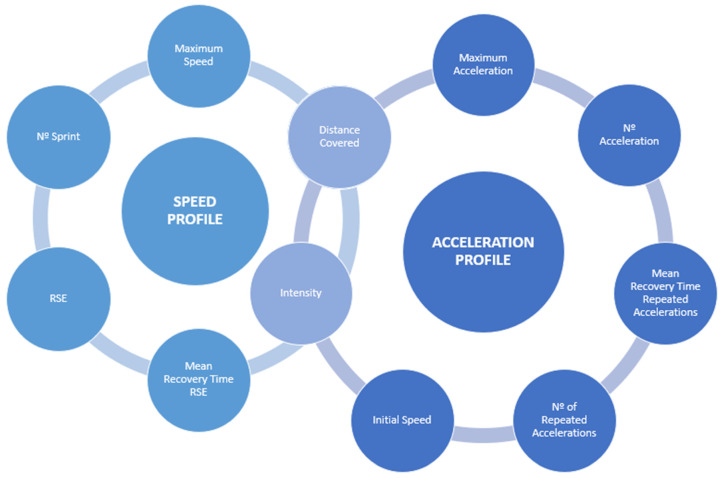
Variables of the Speed and Acceleration Profile in Football.

External load monitoring is valuable in selecting the appropriate load indicators. However, given the complexity of soccer, it is not possible to assess the impact of a single performance indicator when analyzing external load [4,34]. The most commonly used indicators for quantifying external load in soccer include total distance, distances covered in specific speed zones, number of sprints, maximum speed, number of accelerations-decelerations, and exposure time during training and matches, as we can observe in Figure 2 [4,15,20,35,36]. Several authors link these variables to session duration in minutes, which becomes significant when different exposure times exist, such as in a match or a post-match session. Consequently, it is necessary to prorate this load to the exposure time to account for session density [4,37].

### 1.4. Load Distribution and Injuries

In the updated model of injury etiology, training and match loads contribute, along with intrinsic and extrinsic factors, to the multifactorial model of injury etiology [38]. Research on injury prevention in soccer has focused on the relationships between external and internal load indicators to better understand how to prevent them [39]. It has been demonstrated that training load is a modifiable risk factor for overuse injuries [40]. This information becomes even more relevant considering that overuse injuries, which are prevalent in soccer, are often due to errors in load perception and programming [39].

From a preventive perspective, the current literature has established that the injury rate in soccer is higher during competition, with a greater impact on the lower limb due to overuse injuries (66%), particularly muscle injuries, with hamstrings being the most frequently affected [41,42]. Analyzing injury load and economic cost in professional players from European clubs, hamstring injuries had an injury load of 15.4 days/1000 h with an economic cost of €90,367/1000 h, figures that are significant in terms of club performance [43].

In a recent study, Perez et al. demonstrated the impact of weekly external training load and matches as risk factors for muscle overuse injuries. Current data suggest that the combination of a high external training load during the week and a short high-intensity running period during the match could increase the risk of muscle injuries in professional soccer players [44].

Similar to training load, playing position could substantially influence football players’ injury rates [45]. According to Swart et al., midfielders experienced the highest absolute number of injuries (50%), followed by defenders (33%) and forwards (17%) [46]. Likewise, Leventer et al. found that midfielders suffer the highest number of injuries (38%), followed by defenders (30%) and forwards (21%) [45].

## 2. Speed Profile

### 2.1. Maximum Velocity

Among load indicators, the maximum running speed or peak velocity that a soccer player can reach during a match has become one of the most popular variables for assessing a player’s physical talent [30]. Additionally, optimizing maximum speed enables players to respond more effectively to the demands of the game [4]. It is essential to consider each player’s position, as faster players tend to reach a lower percentage of their Vmax during matches compared to slower players [47,48]. Generally, forwards are faster than defenders, and both are faster than midfielders. Many contextual variables can influence the analysis of individual speed reached in matches, and caution is needed when making inter- or intra-player comparisons [20,30,49].

**Figure 3:**
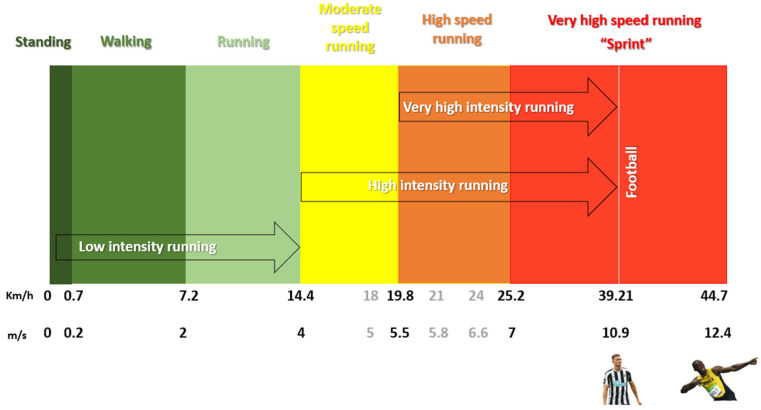
Speed and Intensity Zones: Colored Segments Represent Commonly Used Speed Zones; Arrows Indicate Intensity Zones.

The average maximum speeds reached throughout the season tend to remain stable around 30.7 km/h. Therefore, all teams have players capable of reaching top speeds > 30 km/h, which limits the discriminatory usefulness of maximum speeds to distinguish between higher- and lower-ranked teams. Most players (56%) reached a maximum speed between 32.0 and 33.9 km/h, and only 0.6% of players (three individuals) reached speeds above 35 km/h [30,50]. Besides categorizing players as fast, moderate, or slow with maximum speeds > 32.70 km/h, between 31.70–32.69 km/h, and <31.69 km/h, respectively, another major advantage of maximum speed is its use in defining intensity zones (Figure 3) [20,51].

### 2.2. Absolute Threshold

As shown in Figure 3, player activity is classified into different speed or intensity zones ranging from 0 to 36 km/h, but there are no standardized speed zones. The lack of a universal definition leads to confusion about speed-level thresholds, which can result in erroneous conclusions based on a fixed speed threshold [24,52]. Using a fixed threshold determines absolute speed ranges, that is, arbitrary speed zones independent of players’ fitness levels. Absolute ranges appear to be commonly adopted in soccer; however, interpreting arbitrary speed zones has the disadvantage of masking individual capabilities [20,35,53]. Intensity zones based on absolute ranges are typically divided into six zones, measuring the distance covered according to the speed attained.

- Standing 0–0.6 km/h [34]
- Walking > 0.7–7.2 km/h [34]
- Running > 7.2–14.3 km/h [34]
- Medium-speed running (MSR) 14.4–19.8 km/h [34,49]
- High-speed running (HSR) 19.8–25.1 km/h [10,33,34,49,54,55,56,57,58]
- Sprint or very-high-speed running (VHSR) > 25.2 km/h [10,33,34,49,55,56,57,59]

The lack of consensus in defining absolute thresholds leads to the use of five other predetermined thresholds [52]:

- Walking 0–7 km/h [5,52]
- Running 7–13 km/h [5,52]
- MSR > 13–18 km/h [5,52,60]
- HSR > 18–21 km/h [5,52,60]
- Sprint > 21 km/h [5,52,60]

In a more isolated manner, some authors describe only four zones, using the following thresholds: <6 km/h (low), 6–18 km/h (moderate), 18–24 km/h (high), and >24 km/h (very high). In addition to the described ranges, it is important to analyze the terminology used; some authors employ the term HSR to denote speeds > 14.4 km/h (MSR) and VHSR for speeds > 20 km/h (HSR) [51,61,62].

In soccer, research often focuses on the distance covered at high intensity, and several authors assert that high-intensity actions are considered the best indicator of performance [24,52,54]. Although some studies consider an absolute threshold around 18 km/h to determine the distance covered at high speed, others use a threshold of 19.8 km/h, indicating a clear lack of consensus in the current literature regarding the categorization of these actions [4,10,63,64,65,66]. Some researchers use the term “high intensity” to encompass both high-intensity and sprint segments, combining the distances covered in both ranges, which further complicates potential comparisons among authors [55]. Within high intensity, the distance covered during sprints is even less defined, with differences of more than 4 km/h in the two most commonly used fixed thresholds of 21 and 25.2 km/h; some authors even use a threshold of 24 km/h [8,9,12,64,66,67,68,69]. The lack of clarity in sprint thresholds arises from how they are recorded; they can be counted numerically or by the distance covered. Generally, a sprint is recorded as an effort that involves a minimum movement of 1 m, maintained for at least 1 s, and reaching a defined speed [66]. Therefore, when sprints are recorded numerically, an action can fall into the high-intensity zone (speed > 21 km/h) or very-high-intensity zone (>24 or 25 km/h, depending on the authors) [55]. Confusion arises when a high-intensity threshold is defined as distance covered during a sprint, as it would actually refer to the distance covered at very high intensity [12]. When comparing results across various studies, it is essential to differentiate between the number of sprints at high or very high intensity and the distance covered during sprints, which would be equivalent to the distance covered at very high intensity [70].

### 2.3. RSE and RSA

In the context of speed profiles, performing high-intensity actions intermittently and repeatedly is a key factor for physical performance [71]. The ability to repeat efforts, referred to as “repeated sprint ability” (RSA), is a fitness requirement that quantifies maximal or near-maximal efforts such as sprinting or accelerating, interspersed with brief recovery intervals consisting of either complete rest or low- to moderate-intensity activity [72,73]. Repeated sprint exercise (RSE) and intermittent sprinting differ in recovery times, with almost complete recovery of 60 to 300 s for intermittent sprints and recovery periods of less than 60 s for RSE [74,75]. Buchheit et al. classified high-intensity actions based on recovery duration between repeated efforts, with times of 30 s, 31 to 60 s, and >61 s [76]. Recovery time is a critical factor in the onset of fatigue and has been linked to the ability to reproduce sprints [71,72]. Moreover, the energy cost of intermittent activities is 3.1 to 6.3 times greater than that of running at a constant speed, resulting in increased internal load during intermittent running exercises, such as shuttle runs or near-maximal accelerations [77]. The physiological demands during repeated sprint exercise are primarily affected by the intensity of accelerations [78,79].

Significant differences were found between field positions, with forwards exhibiting significantly better RSA compared to defenders and midfielders. However, no differences were observed in high-intensity activity across positions [80]. Conversely, Carling et al. reported that midfielders performed more high-intensity actions separated by short recovery times (20 s), and running intensity was higher during recovery periods. Regarding full-backs (FB), the number of high-intensity RSEs was statistically greater than in other playing positions [71].

### 2.4. Relative Threshold of Velocity

When the importance and relevance of high-intensity actions in match outcomes have been described, the use of relative speed ranges could address this issue [51,81]. The arbitrary method is commonly employed in professional football to quantify external load data, while the use of individualized methods is on the rise [20]. Utilizing absolute thresholds may underestimate or overestimate the intensity of actions during matches [54,82]. Although activity profiles have been extensively studied, a common methodological limitation is the exclusive use of absolute values. Recent findings suggest that the specific demands of each player should be considered individually [51]. An individualized threshold based on a player’s maximum speed allows for the evaluation of each player’s specific demands, reducing error in quantifying physical performance at different intensities [52]. 

Given the significant variability in running capacity among different players, it is logical to individualize sprint thresholds, high-speed runs, and moderate speeds [51]. This approach of using a relative threshold compared to an absolute threshold could reduce the risk of underestimating or overestimating the players’ effort, as it has been shown that the distance covered can be misinterpreted based on the maximum speed each player is capable of achieving [51,52]. To establish relative thresholds, it is essential to consider each player’s maximum speed, enabling the calculation of thresholds as percentages based on their attained Vmax (Table 2) [68,83,84]. In 2015, Reardon et al. set a value of 60% of maximum speed to define a high-intensity threshold [48]. This was further supported and expanded in 2016 by Castellano et al., who described three relative thresholds: >40% of maximum speed for low intensity, 40-60% for moderate intensity, and >60% for high intensity [85]. Subsequently, even more refined relative thresholds were defined based on intensity: low (0% to 19.99%), moderate (20% to 54.99%), high (55% to 74.99%), and sprint (>75%). These relative zones correspond to the previously defined absolute intensity zones: low (<6 km/h), moderate (6–18 km/h), high intensity (18–24 km/h), and sprint (>24 km/h), thereby adapting to each player’s individual capabilities [51]. Similar thresholds were used for youth football players to define speed and intensity zones: low < 34%, moderate 34–61%, and high > 61% [86].

### 2.5. Comparison Relative and Absolute Threshold

The high-intensity running distance is significantly overestimated in faster players when compared with their relative thresholds. Similarly, in slower players, the high-intensity distance is underestimated relative to their own thresholds [51,87]. Faster players can operate at a relatively lower percentage of their maximum capacity compared to slower players, who may be performing at a relatively higher percentage of their maximum. Likewise, sprint distance is overestimated for faster players, while it is underestimated for slower players [51]. This is because, for a very fast player capable of reaching a maximum speed of 36 km/h, attaining a speed of 24 km/h is less demanding than for a player with a maximum speed of 31 km/h. Thus, it is easier for the faster player to accumulate distance above that speed than for a slower player, a discrepancy not present with a relative threshold, as it adapts to each player’s individual capability. According to Gabbett et al., comparing positions using an absolute HSR threshold (>21 km/h) versus a relative threshold (>60% Vmax), forwards can cover a distance of 269 m or 354 m, respectively. In contrast, defenders cover 697 m or 570 m, respectively [87]. Therefore, high-intensity distance can vary between 3% and 5% of the total distance covered, depending on the chosen threshold, which is an important consideration when high intensity represents 2% to 15% of total distance [48,51,87].

If there is a discrepancy between the absolute and relative quantification of workload, such a discrepancy will have significant implications for planning individualized training programs. It is essential to accurately quantify each individual’s workload, relative stress, and recovery status to achieve an effective training program [51]. So far, studies have not provided a rationale for the use of an absolute threshold over a relative threshold [52]. In conclusion, for any intensity range, an individualized threshold based on the maximum speed a player can reach could be more specific and precise for assessing physical demands than an absolute threshold [47,52,54]. In addition to the chosen threshold, a soccer player’s workload indicators may vary according to age [76], position [13,67,76,88] and the accumulated fatigue during the match [63,89].

### 2.6. According to Playing Position

It is important to remember that soccer players have individual roles within the team, as each has specific tactical tasks and distinct physical needs during matches [10,47]. There are countless positional possibilities depending on the tactical model adopted by the coach. However, positions are generally grouped for programming purposes into goalkeepers (GK), central defenders (CD), full-backs (FB), central midfielders (MF), wide midfielders (WMF), and forwards (FW) (Table 3) [8,31,67,90,91].

Currently, there are divergences regarding the influence of the tactical system and the individual demands of each playing position. Recent studies have shown that FW and MF experienced greater physical demands when playing in a 1-4-2-3-1 tactical formation compared to a 1-4-4-2 formation [92]. In a comparison of eight different formations, the results revealed that the extent to which tactical formation affects match performance depends on the position. In terms of physical performance, CDs and FBs showed greater sprint distances when playing in a formation with only three defenders at the back (1-3-4-3, 1-3-5-2) compared to all other formations [93].

Conversely, Bradley et al. found no significant differences in high-intensity distance covered between the 1-4-4-2, 1-4-3-3, and 1-4-5-1 formations. However, an interesting finding was that FW performed 30% more high-intensity running in a 1-4-3-3 tactical formation compared to the 1-4-4-2 and 1-4-5-1 formations [94]. Higher high-speed distance was observed when the match was tied for midfielders, when losing for defenders, and when winning for attackers [85]. Bradley and Noakes similarly reported 17% less high-intensity running for defenders and 15% more for FW in won matches compared to lost ones [95]. Additionally, a Bundesliga study found that the likelihood of winning a match increased by 31.7% when midfielders increased their sprint distance by 100 m (>24 km/h). For FB, increasing the number of sprints improved the probability of winning a match by 8.6% [96]. While this is a reductionist approach that does not reflect the complexity of all game characteristics, it highlights the influence of playing position on multiple physical and technical variables for players [97,98,99].

Regarding high-speed performance across different playing positions, research indicates that CDs engage in considerably less high-speed activity compared to other positional roles (excluding goalkeepers) [70]. Numerous authors support these findings, reporting that CDs perform fewer sprints than any other position [70,100,101,102,103]. In addition to performing fewer high-intensity actions, CDs generally accumulate the lowest total distance [10,25,70,104,105]. Using relative thresholds, Javier et al. found similar results, with distances between 30–60% and 70–80% of Vmax lower than other positions [52]. In terms of low-intensity time, it accounts for 74.9% to 79.6% of total time based on playing position, with CDs and FWs spending the most time walking or jogging [67,100].

Similarities exist between CD and FW profiles, with FWs running less frequently than CDs [70,100,101,102,103] yet often covering 10% more distance between 60–80% of Vmax than other positions [52]. Redwood-Brown et al. reported that FWs typically cover more high-intensity or sprint distances than defenders and, in some cases, midfielders. However, no significant differences were found between playing positions for high-intensity or sprint distance [106]. Thus, there is evidence that FWs cover less distance in low- or medium-intensity actions, although some authors found no significant differences in high-intensity match demands [105,106]. Considering total weekly load, FWs cover a total distance of 20,330 m and FB 17,862 m, with no significant differences between positions in terms of weekly total distance covered [107].

During training sessions, wide players (FB and WMF) and FW cover the most high-intensity distance, which is consistent with match patterns [107]. It is well-documented that wide players, whether defenders or midfielders, accumulate greater high-intensity and sprint distances than central players [47,48,56,67,70,100,101,102,103,104,108]. Recent studies reveal that WMFs spend more time in high-intensity zones, covering greater distances between 18–21 km/h and >21 km/h than all other positions [52,109]. Many other authors define FB as the position that covers the most sprint and high-speed distance (>19.6 km/h), whereas MF is the position that accumulates the least distance at these intensities [47,64,67,100,104].

Overall, MFs cover the highest absolute and relative total distances, achieving greater low-intensity activities and a higher number of efforts, yet accumulating less high-speed and sprint distances compared to other positions [25,34,47,48,52,109]. In terms of total distance covered, the literature is consistent in identifying MFs as those who cover the greatest distance [70,100,101,102,103,105]. They cover double the distance of CDs [10,25,104] and cover more meters per minute than FWs or defenders, both at home and away matches [106]. Their performance is characterized by high total distance, particularly at moderate speeds such as jogging and running [67,100,110].

Regarding maximum speed, offensive players were the fastest, with maximum speeds of 30.6 km/h for FW and WMF [70]. Recent studies have found similar results, with higher speed peaks for WMFs, but the attained speed was higher, between 8.82 and 8.88 m·s^−1^ (31.75 km/h and 31.96 km/h). The slowest players were MFs, with a maximum speed of 7.96 m·s^−1^ (28.65 km/h) [90,111]. Regarding average speed during matches, MFs or WMFs showed significantly higher values [56].

Physical requirements are specific to each playing position, and players develop their profiles according to these positional demands, which may explain the variability in speed profiles by position [90,112]. This implies that improving team success requires a higher level of physical activity in certain positions and greater technical activity in others. Furthermore, the interpretation of speed profile variables during matches must consider the influence of contextual, environmental, or situational factors, such as match location, opponent quality, and match result [98,113,114,115,116,117].

## 3. Acceleration Profile

Interest in accelerometry variables has been growing over the years, and in professional football, they are now considered some of the most commonly used metrics for monitoring players [35,118,119]. This shift in focus may be explained by the fact that players rarely have the time and space to reach maximum speeds and therefore rely heavily on their ability to accelerate maximally [120]. For a more valid measurement of workload, it is essential to include accelerometry-related parameters, such as distance, time, or the number of actions across various zones, as these provide complementary information to the more commonly used speed profile variables [121,122].

The incorporation of accelerometry-related factors into workload monitoring has highlighted a 6% to 10% difference in workload estimation compared to monitoring techniques that rely solely on speed-based metrics [123,124,125,126].

In football, having a greater acceleration capacity can be decisive in critical moments, and it is estimated that during a match, a player performs between 1000 and 1400 short actions, including changes in direction and intensity, approximately every 60 s. This represents about 7% to 10% of the player’s total workload [101,118,127,128]. During matches, English players perform around 656 accelerations, Croatian players around 600, while Spanish players accumulate 581 [31,67,121]. Other studies have found a total of 76 accelerations [126] and 115 accelerations [118]. The variation in methods, tracking systems, and the classification of accelerations makes it challenging to conclude the potential reasons behind these differences [126].

### 3.1. Absolute Threshold and Initial Velocity

To account for the total number of accelerations performed by a player, changes in speed greater than 0.5 m·s^−2^ are generally quantified without differentiating the intensity of each effort [31,100,121]. One of the methods for classifying accelerometry is the absolute method, which categorizes the intensity of the effort based on a predetermined fixed threshold (Table 4).

Varley et al. defined a single threshold > 2.78 m·s^−2^ to classify accelerations as maximum [118]. Other authors used a similar threshold (>3 m·s^−2^) to classify both high-intensity accelerations and decelerations, expanding the terminology with a low threshold for accelerations between 1 and 2 m·s^−2^ and a moderate threshold for accelerations between 2 and 3 m·s^−2^ [34,70,101,121,123,128,129,130]. Isolated studies have reported other thresholds for high-intensity accelerations above 3.5 m·s^−2^ [65]. Lastly, Bradley et al. used two thresholds, considering moderate accelerations between 2.5 and 4 m·s^−2^ and high-acceleration efforts with a threshold > 4 m·s^−2^ [60,64,131]. Ultimately, there is no consensus on defining the absolute threshold for high-intensity accelerations, with data ranging from 2.78, 3, 3.5, and even 4 m·s^−2^ [31,55,56,60,64,65,123,128].

### 3.2. Initial Velocity

**Figure 4:**
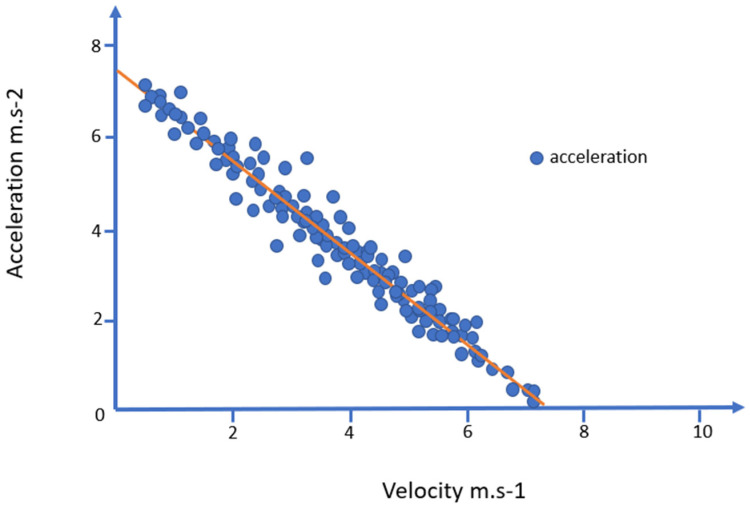
Acceleration Intensity Based on Initial Velocity.

However, this approach does not consider that the ability to accelerate largely depends on the player’s initial velocity (Vini), with a correlation coefficient of 0.98 between the two variables [34]. As illustrated in Figure 4, the maximum possible acceleration for each player progressively decreases as the initial running speed increases [111,132] and most efforts involving high accelerations reach low or moderate peak speeds [34]. Similarly, Aughey and Varley demonstrated that 85% of accelerations do not exceed speeds of 15.84 km/h, and 98% of maximum accelerations (ACCmax) occur from a standstill or at speeds below 14.4 km/h [118]. De Hoyo et al. found that over two-thirds of high-intensity accelerations reach peak speeds below 19.8 km/h, while previous research reported that 40% reach speeds between 7 and 15 km/h, and high-intensity accelerations that end at sprint speeds account for 19% [133].

It is common to mistakenly categorize acceleration as low or high intensity based solely on an absolute numerical value, without considering the initial velocity, which does not always accurately reflect the actual intensity of the effort. When using absolute acceleration thresholds, accelerations starting at higher running speeds can be misclassified [132]. Energy and muscle loads are underestimated when efforts begin at a relatively high initial running speed, classifying an acceleration as low intensity when it is actually high intensity [134]. For example, efforts starting from 16.7 km/h have maximum accelerations of 2.29 m·s^−2^ and would therefore be classified with an absolute threshold of 3 m·s^−2^ as a submaximal effort, despite being very demanding. Indeed, when starting a running effort, few players can reach accelerations of 3 m·s^−2^ [111,132]. Conversely, actions with a low starting speed are overestimated, as an acceleration of 3 m·s^−2^ represents only 50% of the maximum acceleration when starting from a standstill [124]. Ultimately, it is correct to consider the initial speed when categorizing an acceleration as high or low intensity, but it is necessary to go further by using different absolute thresholds based on the initial speed [34,132]. Subsequently, absolute thresholds for high-intensity accelerations based on initial running speed were defined: above 4.51 m·s^−2^ from a standstill, >3.25 m·s^−2^ from walking, >2.4 m·s^−2^ from jogging, >1.72 m·s^−2^ from running [132]. As soccer players are often in motion before initiating an acceleration effort, to anticipate a game situation or follow an opponent, it seems logical to find a significant number of efforts starting from a speed that exceeds being stationary or walking [34]. All of this indicates that the ability to accelerate decreases as running speed increases, and maximum acceleration occurs at the beginning of the action. For these reasons, it is important to use relative thresholds that take into account the initial speed and each player’s individual acceleration capabilities [132,133].

### 3.3. Relative Threshold of Acceleration

To date, the classification of acceleration data based on movement intensity has primarily relied on previously cited absolute thresholds. While the use of these thresholds allows for comparison of physical performance across different cross-sectional and longitudinal studies, their main disadvantage is that they do not take into account the player’s relative individual capacity [120,124,135].

The percentage acceleration method classifies the intensity of an effort based on the ratio of the measured acceleration of that specific effort to the maximum acceleration the individual can achieve (Table 4) [132]. Sonderegger et al. propose four different intensity zones: a high-intensity zone with accelerations > 75% of ACCmax, a moderate-intensity zone with accelerations between 50–75% of ACCmax, a low-intensity zone with accelerations between 25–50% of ACCmax, and a very-low-intensity zone with accelerations below 25% of ACCmax [34]. Using an absolute threshold of >3 m·s^−2^, another of >4 m·s^−2^, and finally a relative threshold of > 75% ACCmax, they found a number of high-intensity accelerations of 120, 59, and 84, respectively [34]. Additionally, these relative thresholds can be applied based on initial speed across three ranges: from walking (0–7 km/h), from jogging (7.1–14.3 km/h), and from running (>14.4 km/h). According to initial speed, the ACCmax values reached were 6.01 m·s^−2^ from a standstill, 4.33 m·s^−2^ while walking, 3.20 m·s^−2^ while jogging, and 2.29 m·s^−2^ while running [34,132,133].

Ultimately, the relative method could avoid the biases introduced by absolute methods, and calculating intensity thresholds based on individual results would be more convenient for counting and categorizing accelerations [132]. Additionally, the acceleration percentage allows for the determination of individual intensity thresholds specific to a single player or a playing position [34,133].

### 3.4. Position and Intensity

Analyzing the initial speed and acceleration intensity by playing position, Oliva-Lozano et al. (2020) found that initial speed was significantly higher for low-intensity accelerations compared to high-intensity accelerations only for WMF and FW positions. No significant differences were found in initial speed and acceleration intensity for CD, FB, and MF. Therefore, it is essential to evaluate the acceleration profile by playing position, as it influences acceleration intensity and initial speed [111].

Previous studies have shown significant differences between playing positions and maximum acceleration capacity [90,111,133,136]. In general, footballers with higher maximum acceleration rates can jump higher, run faster (over short distances), and achieve changes of direction at higher speeds [137].

When total accelerations are counted, the most commonly used threshold in current literature refers to an intensity > 0.5 m·s^−2^ [31,100]. During matches, the highest number of accelerations was found for CDs with 743 total accelerations, while FWs recorded the lowest with 610 accelerations. However, analysis of the total weekly number of accelerations in training showed no differences between the different playing positions [100]. Using the same threshold, Sekulic et al. found 517 accelerations for midfielders and 451 for FWs, again the position with the fewest accelerations. The total number of accelerations does not consider the intensity or initial speed of each acceleration, preventing an accurate interpretation of the actual load represented by these accelerations [31].

CDs have more accelerations in low (1–2 m·s^−2^) and moderate (2–3 m·s^−2^) thresholds; however, MFs covered the most distance accelerating within the low-intensity threshold compared to CDs and FWs. FBs and WMFs had an acceleration density 10 to 20% higher than central positions (MFs and CDs) [138].

Using an absolute high-intensity threshold of >2.78 m·s^−2^, Mallo et al. found that CDs recorded the highest number of accelerations, while FWs had the fewest [67]. The most commonly used threshold for high-intensity accelerations is an absolute value of >3 m·s^−2^. FBs and FWs were the positions that recorded the highest number of accelerations at this intensity, averaging seven and six accelerations per match, respectively. The position with the fewest high-intensity accelerations was midfielders, with an average of 1.9 accelerations [56,100]. Using the same threshold, Sekulic et al. found similar results, with FWs performing the most accelerations, totaling 39. Other positions recorded around 20 maximum accelerations per match [31]. Alonso-Callejo et al. were the only authors to find higher values for acceleration-related variables in CDs, while the lowest ACCmax values on match day were observed for WMFs [90].

Oliva-Lozano et al. defined WMF as the most demanding position for acceleration profiles, with 34.9 high-intensity accelerations (>3 m·s^−2^), 36 m covered while accelerating, and a maximum acceleration of 4.7 m·s^−2^, WMF covered the most distance accelerating, reached the highest maximum acceleration, and performed the greatest number of accelerations compared to other positions. On the other hand, MF covered the least distance (260 m), had the lowest maximum acceleration (4.4 m·s^−2^), and performed the fewest high-intensity accelerations. Regarding accelerations of intensity < 3 m·s^−2^ MF had the highest number of actions [111].

By analyzing three acceleration-intensity thresholds (low, moderate, high) simultaneously, Barrera et al. suggest that the number of accelerations performed at different speeds varies according to the positional demands. For game roles, the highest number of low-intensity accelerations (1–1.9 m·s^−2^ was performed by MFs, who had substantially higher values compared to all other positions. For moderate-intensity accelerations (2–2.9 m·s^−2^), attacking players (OAs) performed the most actions, showing differences with all positions except FBs, who also differed from CDs, FWs and MFs. For high-intensity accelerations (3–4.0 m·s^−2^), OAs and FWs had the highest performance, significantly different from other positions (MFs, CDs, and FBs) [70].

Using a relative threshold of 70% of maximum acceleration, CDs had fewer accelerations, while lateral positions (FBs and WMFs) accelerated more often than any other position [126]. De Hoyo et al. took it a step further with a relative acceleration profile based on initial speed. De Hoyo et al. went further by analyzing relative acceleration profiles based on initial speed. With an intensity of 75% of maximum acceleration (high intensity), CDs showed more accelerations from walking (0–7 km/h) compared to jogging (7.1–14.3 km/h). FWs and WMFs accelerated more from running (>14.4 km/h) than from walking or jogging. FBs completed more accelerations from walking and running than from jogging. Finally, MFs performed a greater number of high-intensity accelerations from walking, and their accelerations from running were greater than from jogging. Comparing the total number of high-intensity accelerations regardless of initial speed, FW, WMF, and FB performed more accelerations than CD and MF. Additionally, MF performed more high-intensity accelerations than CD [133].

With an intensity threshold of >2 m·s^−2^, wide players accelerated significantly more than central players, but these results were only found during the first half of the game, with no differences in the second half or during a full match. Therefore, the ability to accelerate depends on the position and the microcycle day [90,108].

The theoretical maximum acceleration ranged from 5.73 m·s^−2^ for FBs on the day before the match to 8.68 m·s^−2^ for CDs on match day, while the recorded maximum acceleration was 3.27 m·s^−2^ for MFs the day before the match and 5.35 m·s^−2^ for CDs on match day. Both theoretical and recorded data agreed that the least intense day was the day before the match, and the most intense was match day. Additionally, the recorded data showed that the most intense training day in terms of acceleration throughout the week was the first loading day (Wednesday), except for MFs, for whom it was Thursday [90]. Stevens et al. found similar data, with the most intense acceleration load occurring on Wednesday, close to matching the game load, with a 90% overlap [122]. This highlights the importance of recording accelerations during both matches and training [4].

### 3.5. RAA and RHAA

The ability to repeat accelerations, known as “repeated acceleration ability” (RAA), is defined as the capacity to accelerate repeatedly (three or more accelerations) with short recovery times (less than 45 s) [139]. RAA has been proposed as an alternative physical capability that may be more relevant to performance than repeated sprint ability (RSA), since high-intensity accelerations require significant energy expenditure and are up to eight times more frequent than sprints. The results showed that RAA profiles were relatively homogeneous, with no significant differences between playing positions or between different parts of the game [140]. The use of absolute thresholds of 1.5 m·s^−2^ to define RAA efforts might not accurately reflect what happens during matches, as this threshold could be too low and might overestimate high-intensity runs. The ability to repeat submaximal efforts may not be as critical for performance, and several authors have defined the ability to repeat high-intensity accelerations (RHAA) [139,140,141]. RHAA is defined as a minimum of three consecutive high-intensity actions with an average recovery duration of 20 s or less between efforts [71]. High-intensity accelerations were measured using relative thresholds of 70% and 80% of the ACCmax obtained during a 40-m sprint test. An average of eight RHAA efforts were detected with a 70% threshold and 5.1 with an 80% threshold. The average number of efforts within each RHAA was four and 3.6 for the 70% and 80% thresholds, respectively [139].

Regarding RHAA by playing position and game timing, there was a slight decrease in the average number of RHAA efforts in the second half for all positions except midfielders (MF), using a 70% ACCmax threshold. With a higher threshold (80% ACCmax), midfielders showed a moderate decrease in RHAA in the second half, with no significant effects for FWs, FBs, or WMFs. For wide players (FBs and WMFs), a longer RHAA effort duration was recorded compared to other positions [139]. In conclusion, RHAA occurs frequently in football, with small but significant differences between playing positions, as well as between the first and second halves of the game [139,140].

## 4. Conclusions and Future Directions

There is no evident consensus due to the lack of homogeneity in intensity thresholds. Furthermore, the relationships between the two profiles are unclear; faster players do not necessarily achieve the maximum acceleration values [90,111,133]. Monitoring acceleration and velocity profiles more comprehensively not only appears important from a training load and injury-prevention perspective but also provides the coaching staff with specific information necessary to develop and prescribe training protocols that are replicable to match demands [120]. The abundance of external load measures requires a thorough selection of the most useful variables for the specific demands of each playing position. Standardizing the classification of these various measures is of vital importance for organizing this task, as well as when attempting to compare the results obtained in different studies [56].

The use of GPS devices in football players’ daily routines provides a virtually inexhaustible source of individualized data. Their emergence is in response to the growing need to monitor training loads, prevent overload and fatigue, and identify and combat the most common muscular injuries among football players. GPS devices also allow for the assessment of an individual player’s response in various positions on the field. These results can assist sports scientists, medical staff, and coaches in understanding the variability of relative speed and acceleration profiles, thereby aiding the design of individualized training programs tailored to the positional demands of each player.

When referring to an “individualized” load quantification, a methodology based on relative thresholds adapted to the player’s maximum physical capacities should be applied, rather than merely categorizing variables by playing position. Additionally, relative individualization uses percentages that could enable direct comparison of external load variables across different devices. This data could be even more relevant for accelerometry variables, given the variability that exists between devices or different brands.

The positional differences between the speed profile and acceleration profile are distinct; therefore, it is recommended to collect variables from both profiles. In addition to quantifying across various intensities, it is suggested to quantify at least the number of actions performed and the distance covered for both profiles, as discrepancies exist between these variables depending on the playing position. The initial speed should also be taken into account when assessing the acceleration profile.

Quantifying these variables using relative thresholds in both profiles could also be highly beneficial throughout the entire rehabilitation process of an injured player. During the gradual return of the player to the field, physiotherapists and physical trainers alike often question whether the player is ready to engage in group training sessions and subsequently compete. Measuring external load in an individualized manner, based on the player’s current maximum physical capacities, could optimize load quantification at each stage of rehabilitation, aiming to ensure performance and minimize the risk of recurrence or relapse. The initial group sessions are part of the reconditioning process, and the player’s maximum acceleration or speed capacities are not yet fully restored. Therefore, each session will be more demanding for the player, and the use of absolute thresholds or the lack of consideration for the initial acceleration speeds could lead to erroneous load quantification, underestimating the actual external load of the session and increasing the risk of recurrence. Additionally, controlling training load and preventing injuries are essential components in promoting public health and well-being. Maintaining an appropriate training load helps to improve physical condition without overburdening the body, which reduces the risk of injuries and contributes to both physical and mental well-being. This practice not only protects athletes but also benefits anyone engaging in physical activity, from recreational participants to those with specific health goals.

In the context of public health, preventing injuries through controlled training reduces the incidence of musculoskeletal issues, which are a leading cause of work absenteeism and medical expenses. This means that proper management of exercise load not only prevents individual health problems but also optimizes healthcare resources, reducing the demand for medical services and promoting a more active, healthy society.

Furthermore, focusing on injury prevention and load management fosters a culture of safe and sustainable physical activity, which enhances overall population well-being by making exercise accessible and safe. Our results could provide male football players with reference information on the maximum physical capacities to achieve before returning to training or competition. 

Thus, the most relevant studies related to Variables and Playing Position for Velocity and Acceleration Profiles in Football will be detailed in Table 5 for better understanding and clarity.

## Figures and Tables

**Table 1:** An Overview of Tracking Systems in Soccer: Technologies, Models, and Applications in Performance Monitoring.

TRACKING SYSTEMS			
Instrument	Model	Frequency	Nº of Papers
GPS	WIMU PRO™	18 Hz	1
	WIMU PRO™	10 Hz	2
	Catapult OptimEye S5	10 Hz	2
	Catapult Vector S7	10 Hz	4
	Catapult Player Tek	10 Hz	1
	GPSports SPI PRO X	15 Hz	3
	GPSports SPI PRO X II	15 Hz	2
	GPSports SPI HPU	15 Hz	1
	GPSports SPI Elite	1 Hz	1
	STATSports Apex	18 Hz	2
	STATSports Apex ProSeries	10 Hz	2
	STATSports Viper	10 Hz	4
	GPEXE Pro	18 Hz	1
LPM	Inmotiotec GmbH	45 Hz	1
OTS	Second Spectrum^®^	25 Hz	2
	Mediacoach	ND	1
	TRACAB	ND	1
	InStat Fitness	25 Hz	1
RFID	RadioEye™	40 Hz	1
Video Analysis	ProZone Version 3.0	-	2

GPS global positioning system, LPM local position measurement, OTS optical tracking system, RFID radio frequency identification.

**Table 2:** Speed and Intensity Zones in Football: Absolute and Relative Threshold with Corresponding Literature Frequency.

SPEED PROFILE			
	Absolute	Relative	Nº of Papers
Very-Low-Speed Walking	0–5.9 km/h		1
Standing	0–0.7 km/h		1
	0–0.6 km/h		3
Walking	<7.1 km/h		2
	0.7–7.2 km/h		4
	0.1–8 km/h		1
Low-Speed Walking	6–11.9 km/h		1
Jogging	7.2–14.3 km/h		6
	8.1–13 km/h		1
Low-Speed Jogging	12–13.9 km/h		1
Low-Speed Running	0–10.8 km/h		1
	13.1–16 km/h		1
	<14 km/h		1
Medium-Speed Running	14–17.9 km/h		2
Moderate-Speed Running	>14.4 km/h		1
Intermediate-Speed Running	>10.8–19.8 km/h		1
	16.1–19 km/h		1
Running	14.4–19.8 km/h		10
High-Speed Running	4–5.5 m/s	70% Peak Match Speed	1 + 1
	>5.5 m/s	75% Peak Match Speed	2 + 1
	5.5–7 m/s	>Maximal Aerobic Speed	1 + 2
	18–23.9 km/h	>30% Anaerobic Reserve	1 + 2
	18–21 km/h		1
	19.8–25.2 km/h		11
	19.1–22 km/h		1
	>19.8 km/h		4
Very-High-Speed Running	5.5–7 m/s		1
	21–24 km/h		1
	>25.2 km/h		1
Maximum-Speed Running	>22.1 km/h		1
Sprint	>24 km/h	80% Peak Match Speed	5 + 1
	>25.2 km/h	85% Peak Match Speed	14 + 1
	>7 m/s	90% Peak Match Speed	4 + 1
		>85% Peak Speed	1
Very-Low-Intensity Running	0–7 km/h	<10–20% Peak of velocity	1 + 1
Low-Intensity Running	<14.3 km/h	<40% Maximum Speed	2 + 1
	7–13 km/h	20–40% Peak of velocity	1 + 1
Moderate-Intensity Running	<19.8 km/h	40–60% Maximum Speed	1 + 2
	13–18 km/h		1
High-Intensity Running	>5.5 m/s	>60–75% Maximum Speed	2 + 1
	5.5–7 m/s	60–80% Peak of velocity	1 + 1
	>14.4 km/h		3
	17–23.99 km/h		1
	18–21 km/h		1
	>19.8 km/h		2
Very-High-Intensity Running	>19.8 km/h	>75% Maximum Speed	2 + 1
	>21 km/h	>80% Peak of velocity	1 + 1

**Table 3:** Distribution of Playing Positions, Abbreviations, and Frequency in the Literature.

PLAYING POSITION			
	Position	Abbreviation	Nº of Papers
Defender	Center Back	CB	6
	Central Defender	CD	24
	Full-Back	FB	24
	Extreme Defender	ED	1
	Wide Defender	WD	7
	Wing Back	WB	1
Midfielder	Central Midfielder	CM	18
	Midfielder	MF/MD	12/4
	Wide Midfielder	WMF/WMD/WM	7/2/15
	Offensive Midfielder	OM/OMF	1/3
Forward	Forward	FW/F/FO	17/4/1
	Stricker	S/ST	3/1
	Attacker	A/ATT/AT	1/1/3
	Wide Attacker	WA/W	1/2
	Offensive Attacker	OA	1
	Center Forward	CF	3

**Table 4:** Acceleration Zones in Football: Absolute and Relative Thresholds with Corresponding Literature Frequency.

ACCELERATION PROFILE			
	Absolute	Relative	Nº of Papers
Total Accelerations	>0.5 m·s^−2^		3 + 0
Very-Low-Intensity Accelerations	<1.0 m·s^−2^	<25% ACCmax	1 + 1
Low-Intensity Accelerations	0–1 m·s^−2^	<50% ACCmax	1 + 1
	1.1–1.5 m·s^−2^		1
	1–2 m·s^−2^		2
	>1.5 m·s^−2^		1
	<3 m·s^−2^		1
Moderate-Intensity Accelerations	1.6–2 m·s^−2^	<75% ACCmax	1 + 1
	>2 m·s^−2^		2
	2.1–2.5 m·s^−2^		1
	>2–3 m·s^−2^		2
	2–4 m·s^−2^		4
	>2.5 m·s^−2^		1
Intermediate-Intensity Accelerations	1–2 m·s^−2^		1
High-Intensity Accelerations	>2.78 m·s^−2^	>75% ACCmax	1 + 2
	2–3 m·s^−2^		1
	>3 m·s^−2^		9
	3–4 m·s^−2^		1
	>4 m·s^−2^		4
Maximal Accelerationns	>3 m·s^−2^		1
Total Decelerations	>(−) 0.5 m·s^−2^		3
Low-Intensity Decelerations	(−) 0–1 m·s^−2^		1
	<(−) 3 m·s^−2^		2
	(−) 1–1.9 m·s^−2^		1
Moderate-Intensity Decelerations	>(−) 2 m·s^−2^		1
	(−) 2–2.9 m·s^−2^		1
	(−) 2–4 m·s^−2^		4
Intermediate-Intensity Decelerations	(−) 1–2 m·s^−2^		1
High-Intensity Decelerations	>(−) 3 m·s^−2^		7
	(−) 2–3 m·s^−2^		1
	(−) 3–4 m·s^−2^		1
	>(−) 4 m·s^−2^		3
Maximal Decelerations	>(−) 3 m·s^−2^		1
Initial Running	ND	ND	1
	0–7 km/h		2
	7.1–14 km/h		2
	>14.1 km/h		2

**Table 5:** Summary of Study Characteristics Variables and Playing Position for Velocity and Acceleration Profiles in Football.

**Article**	**Type of Study**	**Aim of Study**	Sample Size	Competition Category	Speed Profile	Acceleration Profile	Term	Threshold	Playing Position	Tracking Systems
Alonso-Callejo 2022 [90]	Observational retrospective study	To analyse the differences in the A–S profile of elite football players induced by playing position and the microcycle day	*n* = 25 elite male football players six consecutive microcycles 2021	Spanish Second division	Maximal theoretical speed (abscissa axis intercept (x) in A–S linear regression) Maximmal speed (m/s) Linear slope. Calculated: -A0/S0	Maximal theoretical acceleration Maximal acceleration (m/s^2^)	S0 Smax AS-slope A0 ACC-max	Absolute	CD (*n* = 5) FB (*n* = 3) MF (*n* = 6) WMF (*n* = 6) FW (*n* = 5)	GPS, WIMU PRO™, RealTrack System SL, Almeria, Spain 18 Hz
Modric 2019 [100]	Observational	To identify associations between RP and GPI in professional soccer players and to compare RP and GPI among soccer playing positions	*n* = 101 professional soccer players 14 matches 2018/2019	Croatian Soccer League	Total distance covered (m) Walking (<7.1 km/h) (m) Jogging (7.2–14.3 km/h) (m) Running (14.4–19.7 km/h) (m) High speed running (19.8–25.1 km/h) (m) Sprinting (≥25.2 km/h) (m)	Total accelerations (>0.5 m/s^2^) (count) High-intensity accelerations (>3 m/s^2^) (count) Total decelerations (<[−]0.5 m/s^2^) (count) High-intensity decelerations (<[−]3 m/s^2^) (count)	HSR HIA HID	Absolute	CD (*n* = 26) FB (*n* = 24) MF (*n* = 33) WMF (*n* = 10) FW (*n* = 8)	GPS, Catapult S5 and X4 devices, Melbourne, Australia. 10 Hz
Modric 2020 [107]	Observational	To examine the position-specific associations between running performance (RP) during the training and match in professional-level male soccer	*n* = 15 professional soccer players, 15 matches, and 75 training sessions	Croatian Soccer League	Total distance covered (m) Low-intensity running (<14.3 km/h) (m) Running (14.4–19.7 km/h) (m) High-speed running (19.8–25.1 km/h) (m) Sprinting (≥25.2 km/h) (m) High-intensity running (>19.8 km/h) (m)	Total accelerations (>0.5 m/s^2^) (count) High-intensity accelerations (>3 m/s^2^) (count) Total decelerations (<[−]0.5 m/s^2^) (count) High-intensity decelerations (<[−]3 m/s^2^) (count)	LIR HSR HIR HIA HID	Absolute	CD (*n* = 22 sessions) FB (*n* = 23 sessions) MF (*n* = 29 sessions) WMF (*n* = 6 sessions) FW (*n* = 12 sessions)	GPS, Optim-Eye S5 & X4, Catapult, Melbourne, Australia 10 Hz
Sekulic 2021 [31]	Observational	To evaluate position-specific match running performance (MRP) to determine the effect of COVID-19 lockdowns on the physical performance of professional football players	*n* = 21 professional football players 17 matches 2019/2020	Croatian Soccer League	Total distance covered (m) Low-intensity running (≤ 14.3 km/h) (m) Running (14.4–19.7 km/h) (m) High-intensity running (≥ 19.8 km/h) (m)	Total accelerations (>0.5 m/s^2^) (count) High-intensity accelerations (>3 m/s^2^) (count) Total decelerations (less than –0.5 m/s^2^) (count) High-intensity decelerations (less than –3 m/s^2^) (count)	LIR HIR HIA HID	Absolute	CD (*n* = 38 sessions) FB (*n* = 20 sessions) MF (*n* = 46 sessions)	GPS, Vector S7, Catapult, Catapult Sports Ltd., Melbourne, Victoria, Australia 10 Hz
Sondereg-ger 2018 [34]	Observational	To investigate the strengths and limitations of different indicators to measure physical load	*n* = 139 junior players (*n* = 70 elite and *n* = 69 sub elite) 14 matches (*n* = 7 elite, *n* = 7 sub elite) and 181 files (*n* = 90 elite, *n* = 91 sub elite)	National under-18 (U18) Switzerland	Total distance (m) Standing (0.0–0.7 km∙h^−1^) (m) Walking (>0.7–7.2 km∙h^−1^) (m) Jogging (>7.2–14.4 km∙h^−1^) (m) Running (>14.4–19.8 km∙h^−1^) (m) High-speed running (>19.8–25.2 km∙h^−1^) (m) Sprinting (>25.2 km∙h^−1^) (m)	Initial running speed (km/h) Low acceleration (>1–2 m∙s^−2^) (nº) Moderate acceleration (>2–3 m∙s^−2^) (nº) High acceleration (>3 m∙s^−2^) (nº) High acceleration (>4 m∙s^−2^) (nº) Very low (<25% amax) (nº) Low (<50% amax) (nº) Moderate (<75% amax) (nº) High (≥75% amax) (nº)	HSR Vinit Amax	Absolute And Relative	CD (*n* = 15, (files *n* = 22) FB (*n* = 18, (files *n* = 24) MF (*n* = 17, (files *n* = 21) WMF (*n* = 7, (files *n* = 8) FW (*n* = 13, (files *n* = 15)	LPM (local position measurement) Inmotiotec GmbH, Regau, Austria 45 Hz
Martínez-Cabrera 2017 [91]	Observational	To compare metabolic power (MP) and the traditional approach using speed running during soccer matches in absolute values and in zones of intensity in function of the playing positions	*n* = 38 professional soccer players 18 friendly matches 2013/2014	Romanian First League	Walking (0.1 to 8 km/h) (m) Jogging (8.1 to 13 km/h) (m) Low-speed running (13.1 to 16 km/h) (m) Intermediate-speed running (16.1 to 19 km/h) (m) High-speed running (19.1 to 22 km/h) (m) Maximum-speed running (>22.1 km·h^−1^) (m)	ND	LSR ISR HSR MSR	Absolute	CD (*n* = 64 files) WD (*n* = 55 files) CM (*n* = 58 files) WA (*n* = 70 files) A (*n* = 53 files)	GPS, GPSports SPI PRO X II, Canberra, Australia 15 Hz
Martínez-Cabrera 2021 [134]	Observational	To analyze the characteristics of acceleration efforts using individual relative thresholds according to the initial speed during official matches in elite young soccer players according to player position	*n* = 26 young soccer players 18 matches (*n* = 108 match files)	Spanish soccer club (La Liga BBVA)	Walking (S1 = 6 km/h), Jogging (S2 = 10.8 km/h), Running (S3 = 15 km/h)	Initial speed (km/h) 0–7 km/h 7.1–14 km/h >14.1 km km/h Acceleration maximum (m·s^−2^) Number of high accelerations (>75% Accmax) Number of high accelerations (>3 m·s^−2^)	Sinit Amax	Absolute And Relative	CD (*n* = 40 files) FB (*n* = 23 files) MD (*n* = 18 files) W-MD (*n* = 20 files) S (*n* = 7 files)	GPS, SPI Pro X; GPSports Canberra, Australia 15 Hz
De Hoyo 2018 [133]	Cross-sectional design	To analyse the acceleration profile in elite professional soccer players according to their initial speed but also considering players’ position	*n* = 24 professional male soccer players 35 competitive matches 2015/2016	Spanish soccer club (La Liga BBVA)	ND	Initial speed (km/h) 0–7 km/h 7.1–14 km/h >14.1 km km/h Maximum acceleration (m·s^−2^) Number of accelerations (>75% Accmax)	Vinit Amax	Relative	CB (*n* = 14 files) FB (*n* = 20 files) MD (*n* = 20 files) W-MD (*n* = 16 files) S (*n* = 11 files)	GPS, SPI Pro X; GPSports Canberra, Australia 15 Hz
Oliva-Lozano 2020 [111]	Observational	To describe positional differences in the acceleration and sprint profiles of professional football players in match-play, and analyse start speeds required based on the intensity of accelerations and decelerations	*n* = 23 professional male football players 30 competitive microcycles	Spanish Second Division (LaLiga 123)	Total sprint actions (above 24 km/h) (count) Total distance covered by sprinting (above 24 km/h) (m) Average distance covered per sprint (above 24 km/h) (m) Maximum speed reached in the match (km/h) Duration of sprint (s)	Total distance covered accelerations (m) Total distance covered decelerations (m) Total number of low-intensity accelerations (below 3 m/s^2^) Total number of high-intensity accelerations (above 3 m/s^2^) Total number of low-intensity decelerations (above −3 m/s^2^) Total number of high-intensity decelerations (below −3 m/s^2^) ACCHIGH—DECHIGH Average magnitude of accelerations (m/s^2^) Average magnitude of decelerations (m/s^2^) Maximum magnitude of accelerations (m/s^2^) Maximum magnitude of decelerations (m/s^2^)	SPA SPD SPD-avg Vmax ACC-dis DEC-dis ACC-low ACC-high DEC-low DEC-high DIFF-acdc ACC-avg DEC-avg ACC-max DEC-max	Absolute	CD (*n* = 4) FB (*n* = 5) MF (*n* = 5) WMF (*n* = 4) FW (*n* = 5)	GPS, WIMU Pro, Real Track Systems, Almería, Spain 10 Hz
Barrera 2021 [70]	Quasi-experimental design	To evaluate the activity profile of different positional roles in competitive professional soccer matches	*n* = 25 professional soccer players 11 official matches 2019/2020	Portugueses LigaPro	Maximum speed (km/h) Total distance (m) Very-low-speed walking (0–5.9 km·h^−1^) (m) Low-speed walking (6–11.9 km·h^−1^) (m) Low-speed jogging (12–13.9 km·h^−1^) (m) Medium-speed running (14–17.9 km·h^−1^) (m) High-speed running (18–23.9 km·h^−1^) (m) Sprinting (24 km·h^−1^) (m)	Number of low acceleration (1.0–1.9 m∙s^−2^) Number of moderate acceleration (2–2.9 m∙s^−2^) Number of high acceleration (3–4 m∙s^−2^) Number of low deceleration (1.0–1.9 m∙s^−2^) Number of moderate deceleration (2–2.9 m∙s^−2^) Number of high decelerations (3–4 m∙s^−2^)	ND	Absolute	CD (*n* = 42) WD (*n* = 31) CM (*n* = 34) OA (*n* = 28) CF (*n* = 14)	GNSS, SPI HPU, GPSports, Canberra, Australia 15 Hz
Arjol-Serrano 2021 [92]	Observational	To examine the differences in the physical demands and technical- tactical actions encountered by soccer players between two playing formations (1–4-2-3-1 and 1-4-4-2) for each playing position	*n* = 23 professional male soccer players 31 official matches	Spanish Second Division	Total distance (m) Distance covered (14.4 km·h^−1^) (m) Distance covered (19.8. km·h^−1^) (m) Distance covered (25.0 km·h^−1^) (m)	Number of accelerations (2–4 m·s^−2^) Number of accelerations (>4 m·s^−2^) Number of decelerations (2–4 m·s^−2^) Number of decelerations (>4 m·s^−2^)	TD Acc Dec	Absolute	CD (*n* = 48 files) WD (*n* = 44 files) CM (*n* = 28 files) WM (*n* = 27 files) OM (*n* = 28 files) FW (*n* = 29 files)	GPS, APEX pod accelerometer, MAPPS Technology and Bluetooth LE; STATSports, Newry, North Ireland 18 Hz
Mallo 2015 [67]	Observational	To examine the physical demands imposed on professional soccer players	17 pre-season friendly matches (*n* = 111 files) 2011/2012–2012/2013	Spanish First Division “La Liga”	Total distance (m) Standing still (0–0.6 km·h^−1^) (m) Walking (0.7–7.1 km·h^−1^) (m) Jogging (7.2–14.3 km·h^−1^) (m) Running (14.4–19.7 km·h^−1^) (m) High-speed running (19.8–25.1 km·h^−1^) (m) Sprinting (>25.1 km·h^−1^) (m) High-intensity running (>14.4 km.h^−1^) (m) Very-high-intensity running (>19.8 km.h^−1^) (m) Maximal running speed (km/h)	Number of accelerations (<1.0 m·s^−2^) Number of accelerations (1.1–1.5 m·s^−2^) Number of maximal accelerations (1.6–2.0 m·s^−2^) Number of accelerations (2.1–2.5 m·s^−2^) Number of accelerations (>2.5 m·s^−2^) Number of accelerations (>2.78 m·s^−2^)	HSD HIR VHIR	Absolute	CD (*n* = 23 files) FB (*n* = 20 files) CM (*n* = 22 files) WM (*n* = 26 files) FW (*n* = 20 files)	GPS, SPI Elite, GPSports Systems, Camberra, Australia 1 Hz.
Coutinho 2024 [112]	Observational	To compare the microcycle load distribution between teams from different competitive levels	*n* = 78 professional outfield football players, 22 training microcycles, three teams 2022/2023	First, Second, and Third Portugal division	Total distance covered (m/min), Running (14.4 km·h^−1^–19.7 km·h^−1^) (m/min) High-speed running (>19.8 km·h^−1^) (m/min) Sprinting distance (>25.2 km·h^−1^) (m/min)	Number of high accelerations (>3 m/s) (counts/min) Number of high decelerations (>3 m/s) (counts/min)	HSR	Absolute	CB (*n* = 16) FB (*n* = 12) MF (*n* = 22) W (*n* = 16) S (*n* = 12)	GPS, Catapult, Vector S7, Catapult Sports, Melbourne, Australia 10 Hz
Martín-García 2018 [10]	Observational	To determine the external load of a football team across playing position and relative to competition for a structured microcycle	*n* = 24 players and 42 training weeks and 37 competitive 2015–2016	Reserve Squad of a Spanish La Liga club	Total (m), High-speed running (>19.8 km/h) (m) Sprint distances (>25.2 km/h) (m)	Number of Accelerations (>3.m·s^−2^) Number of Decelerations (>3 m·s^−2^)	TD HSR SPR ACC DEC	Absolute	CD (*n* = 3) (GPS = 104) FB (*n* = 6) (GPS = 145) MF (*n* = 3) (GPS = 45) OMF (*n* = 5) (GPS = 121) FW (*n* = 7) (GPS = 90)	GPS, Viper Pod, 50 gr, 88 × 33 mm; STATSports Viper; Northern Ireland 10 Hz
Díez 2021 [62]	Observational	To analyse the physical demands and technical-tactical actions for each playing position according to game location and final outcome in professional soccer players	*n* = 21 professional male soccer players 30 official matches 2017/2018	Spanish Second Division	Total distance (m) Moderate speed running distance (>14.4 km/h) (m) High-speed running distance (>19.8 km/h) (m) Sprint distance (>25 km/h) (m)	Number of Accelerations (between 2–4 m·s^−2^) Number of Accelerations (>4 m·s^−2^) Number of Decelerations (between 2–4 m·s^−2^) Number of Decelerations (>4 m·s^−2^)	TD MSR HSR SPR	Absolute	CD (*n* = 5) WD (*n* = 4) MD (*n* = 8) F (*n* = 4)	GPS, APEX pod accelerometer, MAPPS Technology and Bluetooth LE; STATSports; North Ireland 18 Hz
Kavanagh 2024 [84]	Observational	To examine the relationships between high-intensity distances covered above generic and relative speed thresholds in English Premier League (EPL) matches across two consecutive seasons	*n* = 16 elite male soccer players and 38 matches two consecutive seasons 2019–2020/2021–2021	English Premier League	Total distance (m) High-speed running distance (>5.5 m/s) (m) High-intensity running distance (5.5–7 m/s) (m) Sprint distance (>7 m/s) (m) Total distance covered >Maximal Aerobic Speed Distance covered > 85% peak speed (m) Distance > 30% Anaerobic Speed Reserve (m)	ND	TD HSRD HIRD MAS PS ASR	Absolute And Relative	Defender (*n* = 7) Midfielders (*n* = 6) Forwards (*n* = 3)	Optical Tracking System Second Spectrum^®^, Los Angeles, CA, USA 25 Hz
Duthie 2018 [37]	Observational Longitudinal	To examine differences between the peak running speed, acceleration, and metabolic power of elite youth soccer across a range of age levels by position	*n* = 96 Elite junior soccer players 61 games within the 2015, 2016, and 2017 season, for a total of 441 individual match observations	-	Distance covered per unit of time (m·min^−1^)	Absolute instantaneous acceleration (m·s^−2^) (count)	ND	Absolute	Attacker ATT Defender DEF Midfielder MID Wide WIDE	GPS, VIPER Units; STATSports Newry, UK 10 Hz
Kim 2023 [116]	Observational	To establish differences between positions and other contextual factors (match location, match outcome, playing formation, and score line) for both external and internal MIP variables	*n* = 24 male outfield players 31 matches 338 individual match observations	English Football League Championship Academy	Average speed (m·min^−1^) High-speed running (m·min^−1^; 5.5 to 7 m·s^−1^) Sprinting (m·min^−1^; >7 m·s^−1^)	Average acceleration/deceleration (m·s^−2^)	HSR Ave-Acc	Absolute	CD (*n* = 4) (GPS *n* = 52) WD (*n* = 5) (GPS *n* = 54) CM (*n* = 8) (GPS *n* = 89) WM (*n* = 5) (GPS *n* = 54) ST (*n* = 2) (GPS *n* = 28)	GPS, Vector S7, Catapult Innovations, Melbourne, Australia 10 Hz
Miguel 2022 [131]	Observational Cohort Study	To describe and characterize the daily and weekly external load in an amateur soccer team and based on the weighting factors determined by the match reference, compare the external loads between playing positions	*n* = 24 amateur soccer players 19 competitive microcycles 132 individual match observations 2018/2019	Portuguese regional competition	Total distance covered (m) High-speed running distance (4.0–5.5 m/s) (m) Very-high-speed running distance (5.5–7.0 m/s) Sprint distance (>7.0 m/s) (m)	Total number of accelerations “moderate intensity” (2.0–4.0 m/s^2^) Total number of accelerations “high intensity” (>4.0 m/s^2^) Total number of decelerations “moderate intensity” (2.0–4.0 m/s^2^) Total number of accelerations “high intensity” (>4.0 m/s^2^)	TDC HSRD VHSRD SpD MIAcc HIAcc MIDec HIDec	Absolute	CD (*n* = 4) (GPS *n* = 30) FB (*n* = 4) (GPS *n* = 30) CM (*n* = 6) (GPS *n* = 38) WM (*n* = 5) (GPS *n* = 24) F (*n* = 3) (GPS *n* = 10)	GPS, PlayerTek, Catapult Innovations, Melbourne, Australia 10 Hz
Kavanagh 2023 [135]	Retrospective study	To analyze the positional distances covered above generic and individualized speed thresholds within the most demanding phases of match play	*n* = 17 male professional soccer players 76 official league matches 2019–2020/2020–2021	English Premier League	Total distance covered (m) High speed running distance (5.5 m/s) (m) Total distance covered > Maximal Aerobic Speed Sprint distance (7 m/s) (m) Distance > 30% Anaerobic Speed Reserve (m)	ND	HSR MAS ASR	Absolute and Relative	FB (*n* = 4) CD (*n* = 4) CM (*n* = 3) WM (*n* = 3) F (*n* = 3)	Optical Tracking System Second Spectrum^®^, Los Angeles, CA, USA 25 Hz
Casamich-ana 2021 [130]	Observational	To compare weekly accumulative load during the in-season competitive period by professional soccer players according to the amount of time played in official matches (90-min, > 60-min, < 60-min, and 0-min) regarding the players’ position	*n* = 24 professional football players 42 training weeks and 37 official matches 2015–2016	Reserve squad of a Spanish La Liga	Total distance (m) High speed running (>19.8 km·h^−1^) (m) Sprint meters (>25.2 km·h^−1^) (m) High metabolic load distance (>25.5 W·kg^−1^) (m)	Number of accelerations (ACC; >3 m·s^−2^) Number of decelerations (DEC; <−3 m·s^−2^).	TD HSR SPR HMLD ACC DEC	Absolute	FB (GPS *n* = 34) CD (GPS *n* = 26) MF (GPS *n* = 12) OMF (GPS *n* = 30) FW (GPS *n* = 20)	GPS, Viper Pod, 50 g, 88 × 33 mm, STATSports Viper, Northern Ireland 10 Hz
Djaoui 2022 [119]	Observational	To analyse the influence of congested periods of matches on the acceleration (Acc) and deceleration (Dec) profiles of elite soccer players	*n* = 23 elite male professional soccer players 31 official matches 2016 Two consecutive season (March-December)	National Premier League Swiss	Total distance covered (m) Low-speed running (0–10.8 km.h^−1^) (m) Intermediate-speed running (>10.8–19.8 km.h^−1^) High-speed running (>19.8–25.2 km.h^−1^) (m) Sprint (>25.2 km.h^−1^) (m)	Total distance decelerating (m) Maximal Deceleration (<−3 m·s^−2^) (m) High Deceleration (−3 to <−2 m·s^−2^) (m) Intermediate Deceleration (−2 to <−1 m·s^−2^) (m) Low Deceleration (−1 to <0 m·s^−2^) (m) Total distance acccelerating (m) Low Acceleration (>0 to 1 m·s^−2^) (m) Intermediate Acceleration (>1 to 2 m·s^−2^) (m) High Acceleration (>2 to 3 m·s^−2^) (m) Maximal Acceleration (>3 m·s^−2^) (m)	TDC LSR ISR HSR Tdec MDec MAcc HDec HAcc IDec IAcc LDec LAcc	Absolute	CB (*n* = 5) (GPS = 58) FB (*n* = 5) (GPS = 65) CM (*n* = 6) (GPS = 72) WF (*n* = 4) (GPS = 36) CF (*n* = 4) (GPS = 39)	GPS, Viper, STATSports, Ireland 10 Hz
Guerrero-Calderón 2022 [58]	Observational	To compare the training and match load of professional soccer players according to the playing position, and analyse the relationship between the metabolic and running speed metrics	*n* = 30 professional male soccer players *n* = 33 training weeks and *n* = 38 matches 2015–2016	Spanish First Division	Total distance (m) Low-speed running distance (<14 km/h) (m) Medium-speed running distance (14 to 18 km/h) High-speed running distance (18 to 21 km/h) (m) Very-high-speed running distance (21 to 24 km/h) Sprint running distance (>24 km·h^−1^) (m)	Number of accelerations (2 m·s^−2^) Number of decelerations (<2 m·s^−2^)	LSRD MSRD HSRD VHSRD SPD	Absolute	CD (GPS *n* = 89) ED (GPS *n* = 61) CM (GPS *n* = 71) WM (GPS *n* = 76) FO (GPS *n* = 36)	GPS, GPEXE Pro 18, GPEXE, Udine, Italy 18 Hz
Caro 2022 [59]	Observational	To analyse sub-maximum intensity periods (SubMIP’s) manifested by professional soccer players during official matches according to the player position	*n* = 14 professional soccer players, *n* = 247 individual records, during 15 official matches 2019–2020	Azerbaijan Premier League	Total distance (m) High-speed running (>19.8 km/h) (m) Very-high-speed running or sprint (>25.2 km/h) Mean metabolic power metres per minute High metabolic load distance (>25.5 W/kg)	Number of accelerations (>3.m·s^−2^) Number of decelerations (<−3 m·s^−2^) Acceleration density (%)	HSR VHSR HMLD Met-Pow Acc-Dens	Absolute	CD (GPS = 76) WD (GPS = 50) MF (GPS = 36) OMF (GPS = 26) FW (GPS *n* = 59)	GPS, STATSports APEX ProSeries; STATSports, Newry, Northern Ireland 10 Hz
Garcïa-Calvo 2022 [27]	Observational	To examine the Spanish professional soccer players’ high metabolic load distance profile, comparing competitive level and playing positions	*n* = 1321 players *n* = 18,131 individual match observations 2018/2019–2019/2020	First and Second Spanish Professional Soccer Leagues	High Metabolic Load Distance: Distance covered with a power consumption above 25.5 W·kg^−1^ Running at a constant velocity of 5.5 m·s^−1^ or 19.8 km·h^−1^	High Metabolic Load Distance: Accelerations or decelerations (2 to 4 m·s^−2^)	HMLD	Absolute	CB FB CM WM FW	Mediacoach
Forcher 2022 [93]	Observational	To examine to what extent the physical match performance of professional soccer players is both position and player-specific	*n* = 25 players across 15 clubs 25 matchdays *n* = 163 matches 2019–2020	German Bundesliga	Total distance (m) High-intensity distance (17–23.99 km/h) (m) Sprinting distance (>24 km/h) (m)	Number of accelerations (>1.5 s^−2^)	ND	Absolute	CD *n* = 658 WD (*n* = 244 files) WB (*n* = 122 files) CM (*n* = 538 files) WM (*n* = 187 files) FW (*n* = 215 files)	Tracking system TRACAB, Chyron Hego, Melville, NY, USA
Modric 2023 [110]	Observational	To provide a comparative analysis of RP of professional soccer match-play across two highest-level soccer competitions: UCL and WC	Professional soccer players UCL *n* = 244 matches *n* = 20 WC *n* = 581 matches *n* = 55	Union of European Football Associations Champions League AND Fédération Internationale De Football Association World Cup	Total distance (m) High-intensity running (>5.5 m/s) (m)	ND	TD HIR	Absolute	FB (GPS *n* = 189) CD (GPS *n* = 300) CM (GPS *n* = 195) WM (GPS *n* = 87) FW (GPS *n* = 54)	Optical systems: InStat Fitness InStat Limited, Limerick, Republic of Ireland 25 Hz
Silva 2024 [81]	Retrospective study	To analyse the relations and differences between distances covered during official matches of the Portuguese first division, according to specific thresholds	*n* = 20 elite level soccer players *n* = 34 matches (111 observations) 2021–2022	First division of the Portuguese League	Distance covered > 25.2 km/h (m) Distance covered > 70% peak match speed (m) Distance covered > 75% peak match speed (m) Distance covered > 80% peak match speed (m) Distance covered > 85% peak match speed (m) Distance covered > 90% peak match speed (m)	ND	ND	Absolute And Relative	FB (*n* = 3) CD (*n* = 6) CM (*n* = 5) WM (*n* = 4) FW (*n* = 2)	GPS, Catapult Vector S7—Catapult Sports, Melbourne, Australia 10 Hz
Morgans 2023 [117]	Observational	To examine the impact of playing position (PP), match location (ML), and opposition standard (OS) on team and individual acceleration (ACC) and deceleration (DEC) efforts	*n* = 50 elite football players U23 24 matches 2020/21	English Premier Development League	ND	High-intensity acceleration (>+3 m·s^−2^) (count) Highintensity deceleration (<−3 m·s^−2^) (count)	ND	Absolute	CB (GPS *n* = 68) FB (GPS *n* = 24) CM (GPS *n* = 54) WM (GPS *n* = 15) CF (GPS *n* = 27)	GPS, Apex, STATSports Software; version 4.3.8, Northern Ireland, UK 10 Hz
Oliva-Lozano 2023 [36]	Observational	To investigate the periods in which sprints occurred during official matches and analyze these sprints considering the effect of the playing position and different contextual variables	*n* = 20 male soccer players *n* = 252 sprints *n* = 6 matches	Spanish semi-professional club	Maximum velocity (km/h) Starting Velocity (km/h) Distance covered sprinting (>24 km/h (m)	Maximum acceleration (m·s^−2^) Maximum deceleration (m·s^−2^)	Vmax V0 SPD ACC-max DEC-max	Absolute	FB CD MF WMF FW	GPS, WIMU Pro systems RealTrack Systems, Almeria, Spain
Ingebrigts-en 2015 [108]	Observational	To characterise the acceleration and sprint profiles of elite football match play	*n* = 15 professional players (*n* = 101 observations) 15 home game	Norwegian elite football team (Rosenborg FC)	Total distance covered (m) Walking (from 0 to 7.1 km·h^−1^) (m) Jogging (from 7.2 to 14.3 km·h^−1^) (m) Running (from 14.4 to 19.7 km·h^−1^) (m) High-speed running (from 19.8 to 25.2 km·h^−1^) Sprinting (≥25.2 km·h^−1^) (m) Low- and moderate-intensity activities (locomotion <19.8 km·h^−1^) (m) High-intensity activities (locomotion (≥19.8 km·h^−1^) (m)	Number of acceleration (>2 m·s^−2^)	ND	Absolute	CD (*n* = 3) FB (*n* = 4) CM (*n* = 2) WM (*n* = 4) AT (*n* = 2)	Tracking system RadioEye^TM^ technology (ZXY SportTracki-ng AS, Radionor Communications AS, Trondheim, Norway) 40 Hz
Bradley 2009 [69]	Observational	To determine the activity profiles of a large sample of English FA Premier League soccer players and examine high-intensity running during elite-standard soccer matches for players in various playing positions	*n* = 370 players 28 games competitive season 2005/2006	English FA Premier League	Total distance (m) Standing (0–0.6 km/h) (m) Walking (0.7–7.1 km/h) (m) Jogging (7.2–14.3 km/h) (m) Running (14.4–19.7 km/h) (m) High-speed running (19.8–25.1 km/h) (m) Sprinting (>25.1 km/h) (m) High-intensity running (>14.4 km/h) (m) Very-high-intensity running (>19.8 km/h) (m)	ND	HSR HIR VHIR	Absolute	CD (*n* = 92) FB (*n* = 84) CM (*n* = 80) WM (*n* = 52) AT (*n* = 62)	Tracking system ProZone Version 3.0, ProZone Sports Ltd.1, Leeds, UK
Núñez-Sánchez 2017 [52]	Observational	To compare the relative running demands (m·min^−1^), among different soccer players positions, coded by an absolute threshold vs. an individualized threshold based on splits of 10% of peak velocity, during friendly games, with the same tactical system and monitoring with a GPS	*n* = 20 semiprofessional soccer players four friendly matches	Spanish soccer league	Very-low-intensity running (0–7 km·h^−1^) (m) Low-intensity running (7–13 km·h^−1^) (m) Medium-intensity running (13–18 km·h^−1^) (m) High-intensity running (18–21 km·h^−1^) (m) Very-high-intensity running (>21 km·h^−1^) (m) <10% Peak of velocity (m) 10–20% Peak of velocity (m) 20–30% Peak of velocity (m) 30–40% Peak of velocity (m) 40–50% Peak of velocity (m) 50–60% Peak of velocity (m) 60–70% Peak of velocity (m) 70–80% Peak of velocity (m) 80–90% Peak of velocity (m) >90% Peak of velocity (m)	ND	VLIR LIR MIR HIR VHIR PV	Absolute and Relative	CB *n* = 4 FB *n* = 4 CM *n* = 4 WM *n* = 4 F *n* = 4	GPs, SPI-pro W2b, GPSport, Canberra, Australia 15 Hz
Bradley 2013 [95]	Observational	To examine the effects of high (HPBPT) and low-percentage ball possession teams (LPBPT) on physical and technical profiles in elite soccer matches	*n* = 810 players 54 matches	English FA Premier League	Total distance (m) Standing (0–0.6 km/h) (m) Walking (0.7–7.1 km/h) (m) Jogging (7.2–14.3 km/h) (m) Running (14.4–19.7 km/h) (m) High-speed running (19.8–25.1 km/h) (m) Sprinting (>25.1 km/h) (m) High-intensity running (>19.8 km/h) (m)	ND	HIR	Absolute	CD (*n* = 199) FB (*n* = 177) CM (*n* = 191) WM (*n* = 110) AT (*n* = 133)	Tracking system ProZone Version 3.0, ProZone Sports Ltd.1, Leeds, UK

## Data Availability

Data is contained within the article.
